# Molecular Characterization and Expression of Four Aquaporin Genes in *Impatiens walleriana* during Drought Stress and Recovery

**DOI:** 10.3390/plants10010154

**Published:** 2021-01-14

**Authors:** Marija J. Đurić, Angelina R. Subotić, Ljiljana T. Prokić, Milana M. Trifunović-Momčilov, Aleksandar D. Cingel, Milan B. Dragićević, Ana D. Simonović, Snežana M. Milošević

**Affiliations:** 1 Institute for Biological Research “Siniša Stanković”, National Institute of Republic of Serbia, Department of Plant Physiology, University of Belgrade, Bulevar Despota Stefana 142, 11060 Belgrade, Serbia; heroina@ibiss.bg.ac.rs (A.R.S.); milanag@ibiss.bg.ac.rs (M.M.T.-M.); cingel@ibiss.bg.ac.rs (A.D.C.); mdragicevic@ibiss.bg.ac.rs (M.B.D.); ana.simonovic@ibiss.bg.ac.rs (A.D.S.); snezana@ibiss.bg.ac.rs (S.M.M.); 2 Department for Agrochemistry and Plant Physiology, Faculty of Agriculture, University of Belgrade, Nemanjina 6, 11080 Belgrade, Serbia; ljprokic@agrif.bg.ac.rs

**Keywords:** water relations, aquaporins, molecular structure, drought stress, gene expression

## Abstract

Aquaporins comprise a large group of transmembrane proteins responsible for water transport, which is crucial for plant survival under stress conditions. Despite the vital role of aquaporins, nothing is known about this protein family in *Impatiens walleriana,* a commercially important horticultural plant, which is sensitive to drought stress. In the present study, attention is given to the molecular characterization of aquaporins in *I. walleriana* and their expression during drought stress and recovery. We identified four *I. walleriana* aquaporins: IwPIP1;4, IwPIP2;2, IwPIP2;7 and IwTIP4;1. All of them had conserved NPA motifs (Asparagine-Proline-Alanine), transmembrane helices (TMh), pore characteristics, stereochemical properties and tetrameric structure of holoprotein. Drought stress and recovery treatment affected the aquaporins expression in *I. walleriana* leaves, which was up- or downregulated depending on stress intensity. Expression of *IwPIP2;7* was the most affected of all analyzed *I. walleriana* aquaporins. At 15% and 5% soil moisture and recovery from 15% and 5% soil moisture, *IwPIP2;7* expression significantly decreased and increased, respectively. Aquaporins *IwPIP1;4* and *IwTIP4;1* had lower expression in comparison to *IwPIP2;7*, with moderate expression changes in response to drought and recovery, while *IwPIP2;2* expression was of significance only in recovered plants. Insight into the molecular structure of *I. walleriana* aquaporins expanded knowledge about plant aquaporins, while its expression during drought and recovery contributed to *I. walleriana* drought tolerance mechanisms and re-acclimation.

## 1. Introduction

As sessile organisms, plants are exposed to different abiotic and biotic stress factors in their environment [1]. Among abiotic stresses, drought is one of the most important, with detrimental effects on plant growth and development [1]. Drought disrupts the water status in plants and induces resistance mechanisms to maintain an optimal water amount in tissues. One of the plant resistance mechanisms to drought is the increased accumulation of abscisic acid (ABA), which induces rapid stomata closure as well as changes in gene expression leading to the synthesis of osmoprotectants, antioxidant protection components, proteins with a protective role, water transporters and various products of secondary metabolism [2,3]. Among these components, gene expression for water transporters—aquaporins, is often investigated in drought-stressed plants. Aquaporins are transmembrane proteins, which, in addition to water, can also transport O_2_, CO_2_, glycerol, urea, H_2_O_2_, metals and metalloids across the plant membranes [4]. Aquaporins belong to the Major Intrinsic Protein family (MIP; InterPro: IPR000425) and are found in all living organisms except in thermophilic archaea and intracellular bacteria [5,6]. In plants, aquaporins are present in all organs, including roots, stems, leaves, flowers, seeds and fruits. Most of them were found in the plasma membranes and tonoplast, but their presence is also reported in other intracellular membranes [6]. The molecular weight of aquaporins ranges between 23 and 31 kDa, and all contain six transmembrane regions, with N and C terminal ends localized in the cytoplasm [7]. The transmembrane domains are connected by five loops (A–E), of which B and D are localized in the cytoplasm of the cell and A, C and E are extracellular. Loops B and E are hydrophobic and contain highly conserved amino acid repeats of Asparagine-Proline-Alanine, which represent so-called NPA motifs, located opposite on two sides of the membrane [7,8]. NPA motifs with other aromatic/Arg (Ar/R) motif form a pore in the membrane for the passage of water and various molecules and present the most important parts for the functioning of aquaporins [8]. Four combined aquaporin monomers form a holoprotein, whose structure is stabilized by hydrogen bonds and interactions between loops of individual monomers [7,8]. In the aquaporin holoprotein, besides the four individual monomer pores, exists a noticeable central fifth pore that can also be responsible for the transport of water, CO_2_ and other substrates [6].

In higher plants, aquaporins are categorized into five subfamilies: Plasma membrane Intrinsic Proteins (PIPs), Tonoplast Intrinsic Proteins (TIPs), Nodulin-like Intrinsic Proteins (NIPs), Small basic Intrinsic Proteins (SIPs) and X Intrinsic Proteins (XIPs) [6]. Not all aquaporin families are involved in water transport. NIPs, SIPs and XIPs subfamilies have a lower water transport activity and are mainly involved in solute transport [9]. Each subfamily is further divided into different subgroups. PIPs are the largest plant aquaporins subfamily divided into two subgroups, PIP1 and PIP2. These two subgroups include different protein isoforms with different localization and function in water and solute transport across the plant membranes [4]. Members of PIP1 and PIP2 subgroup can also transport other molecules through membranes such as CO_2_, glycerol, H_2_O_2_ and boron [6,10,11]. While PIP2 members are efficient water channels, there is no definitive consensus in the literature regarding the ability of PIP1 members to transport water. In some plant species, they are shown to be efficient water channels, while in some other PIP1 members exhibit relatively low water transport activity [10]. Recent evidence indicates that PIP1 proteins require heterotetramerization with PIP2 to function in water transport [12,13]. TIPs are the most prevalent on vacuole membranes (tonoplast) and are divided into five subgroups (TIP1–TIP5) [6,14,15]. TIPs in the tonoplast serve as regulators of the intracellular water flow, namely for osmotic adjustment and maintenance of cell turgor pressure. Based on the expression of *Nicotiana tabacum* TIP protein in *Xenopus leavis* oocytes, it has been shown that TIPs can also transport glycerol and urea [16] as well as ammonia, hydrogen peroxide and formamide [15,17]. TIPs on the tonoplast are also used as vacuolar markers [15]. Given the wide range of identified aquaporins, their numerous roles in plant growth and development have also been described. Aquaporins are involved in plant responses to abiotic stress and adjusting the transport of water and other molecules in cells according to the physiological state of plants [8,18,19,20].

The genus *Impatiens* (Balsaminaceae) includes more than 1000 species and is one of the largest genera among flowering plants [21]. Although there are some exceptions, most species of the genus *Impatiens* do not tolerate prolonged drought conditions or prolonged exposure to direct sunlight. Therefore, these species are mostly limited in their distribution to wetlands, such as tropical rainforests and places along rivers, streams or swamps [21]. Due to their beautiful appearance and long flowering period, many species of the genus *Impatiens* are grown all over the world as ornamental plants. *Impatiens walleriana* is the most popular among the species of the genus *Impatiens*, with fleshy, succulent leaves and various colors of flowers that are formed from early spring to late autumn [22]. *I. walleriana* is an annual herbaceous plant, like most species of the genus *Impatiens*. The height of the plant ranges between 30 and 70 cm and is very branched. The leaves are spirally arranged, but often at the tips of the shoots there are leaves facing each other, oppositely arranged. The flowers are zygomorphic and exhibit variability in size, shape and color as an adaptation to different pollinators [22,23]. *I. walleriana* is one of the three plant species (in addition to *Impatiens hawkeri* and *Impatiens balsamina*) of the genus *Impatiens* that have been commercially produced in Serbia for many years. The plant has high requirements for the presence of water in the substrate, the lack of which leads to a rapid drop in turgor pressure in the cells and tissue dehydration [22]. In this work, we describe the molecular structure of four aquaporins (IwPIP1;4, IwPIP2;2, IwPIP2;7, IwTIP4;1) and changes in their expression during drought stress and recovery in *I. walleriana* grown ex vitro. These aquaporins were chosen from *I. walleriana* transcriptome due to their predicted belonging to aquaporin subgroups (PIP1, PIP2 and TIP4), whose members are known to participate in water transport. Previously, we described the effects of drought and recovery treatment on growth, physiological, biochemical and molecular responses in *I. walleriana* [3]. The aim of this study was to characterize aquaporins in *I. walleriana* and investigate their expression in leaves in order to evaluate the impact on water transport in drought-stressed and recovered *I. walleriana*. 

## 2. Results

### 2.1. Characteristic of I. walleriana Aquaporin Proteins and Their Predicted Structural Models 

Characteristics of IwPIP1;4 (partial coding sequence), IwPIP2;2, IwPIP2;7, and IwTIP4;1 (complete coding sequence), including nucleotide sequence length, amino acid number, theoretical protein pI (protein Isoelectric Point), II (instability index) and subcellular localization are given in Table 1.

**Figure 1:**
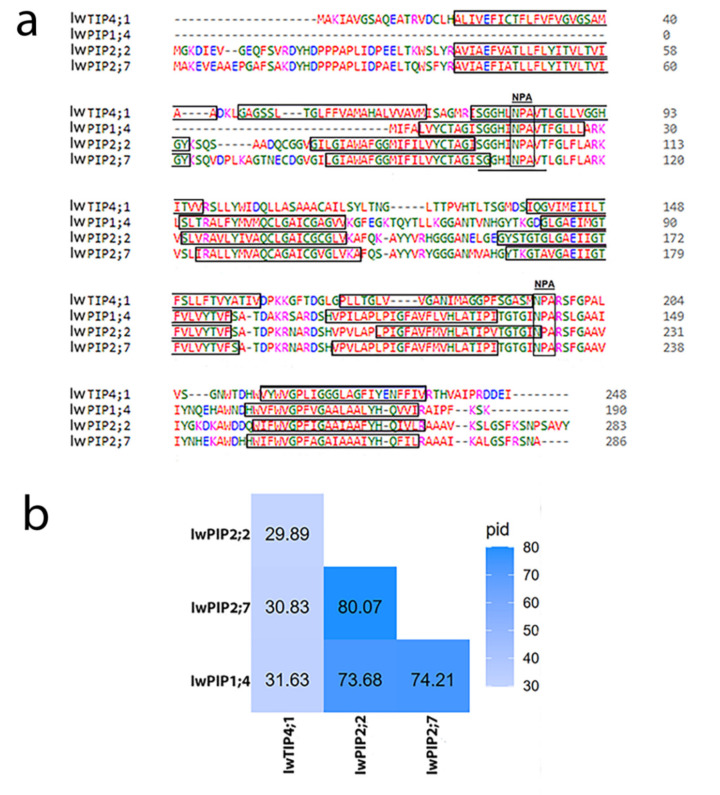
(**a**) Multiple sequences alignment of four *I. walleriana* aquaporins (IwPIP1;4, IwPIP2;2, IwPIP2;7 and IwTIP4;1) constructed using Clustal Omega; inside the square are TMh and NPA motifs, while the Major Intrinsic Protein (MIP) signature sequence is underlined; (**b**) percentage identity heatmap between the *I. walleriana* aquaporins.

**Figure 2:**
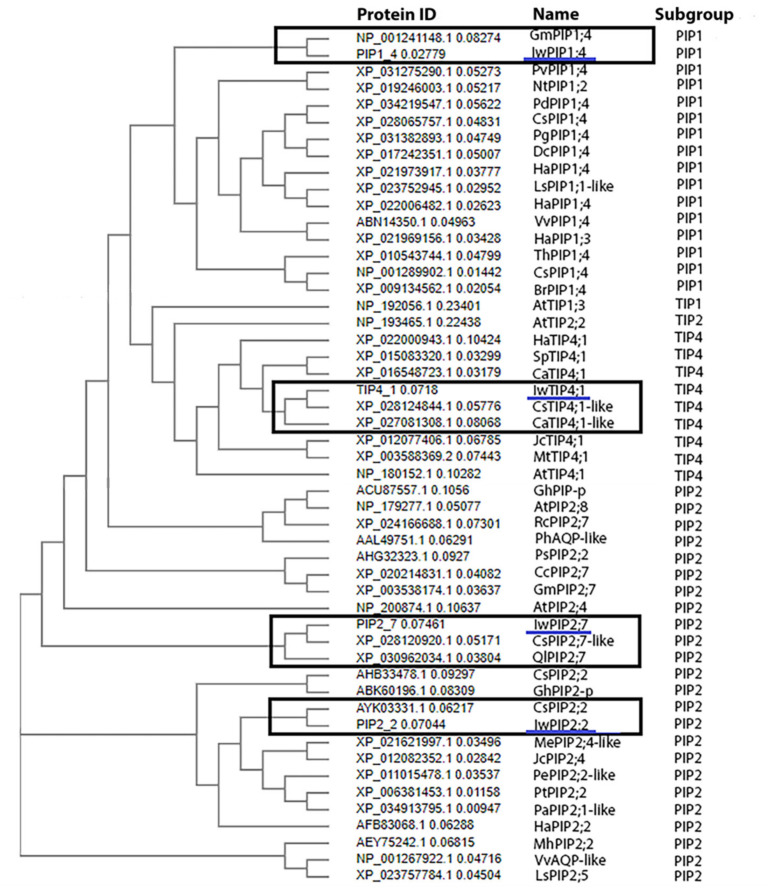
Neighbor-joining phylogenetic tree of *I. walleriana* aquaporins and 47 aquaporins from other plant species constructed based on Clustal Omega multiple sequence alignment.

Multiple alignments for the four analyzed aquaporins from *I. walleriana* are presented in Figure 1a. Conserved NPA motifs are marked, as well as transmembrane helices (TMh) from the TMh1 to TMh6, except for IwPIP1;4, which did not have the TMh1. The signature sequence of aquaporins (SGxHxNPAVT) is underlined in Figure 1a. Protein sequences of IwPIP2;2 and IwPIP2;7 had a similarity of 80.07%. IwPIP1;4 identity with IwPIP2;2 and IwPIP2;7 was 73.68% and 74.21%, respectively, while it shared 31.63% identity with IwTIP4;1. IwPIP2;2 and IwPIP2;7 shared 29.89% and 30.83% of identity with IwTIP4;1, respectively (Figure 1b). Phylogenetic distance between aquaporins from *I. walleriana* and 47 aquaporins from other plants are shown in Figure 2, suggesting that these sequences belong to the PIP1, PIP2 and TIP4 subgroups. Different aquaporin isoforms from other plant species belonging to PIP1, TIP4 and PIP2 subgroups were chosen for phylogenetic distance analysis. Aquaporin IwPIP1;4 shared the most sequence identity with aquaporin GmPIP1;4 from *Glycine max,* while the IwTIP4;1 is the most similar to aquaporins CsTIP4;1-like from *Camellia sinensis* and CaTIP4;1-like from *Coffea arabica*. Aquaporins from the PIP2 subgroup, IwPIP2;2 and IwPIP2;7, are both very similar to appropriate aquaporins from *Camelia sinensis*, while IwPIP2;7 is also closely related to aquaporin QlPIP2;7 from *Quercus lobata.*

**Figure 3:**
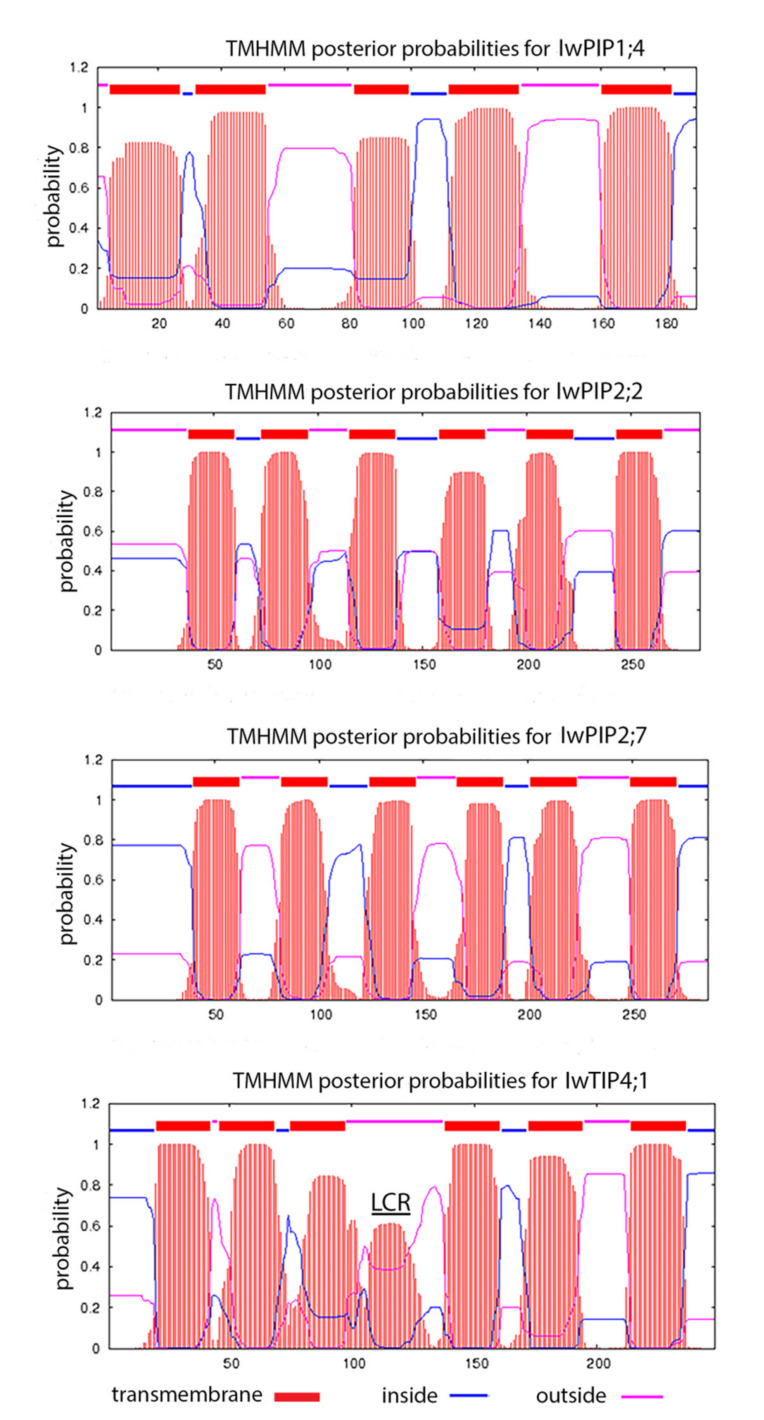
Predicted transmembrane helices (TMh) of four *I. walleriana* aquaporins (IwPIP1;4, IwPIP2;2, IwPIP2;7 and IwTIP4;1) according to TMHMM Server v. 2.0. LCR region in IwTIP4;1 is predicted by SMART program.

The predicted TM helices for all aquaporins are presented in Figure 3. It can be noticed that IwPIP1;4, being a partial sequence lacking N-terminus (Figure 1a), did not have the first TM helix, which corresponded to the lower amino acid number and molecular weight of this protein. The three other aquaporins had six transmembrane regions. IwTIP4;1 had an additional region from 107 to 120 amino acids (“LLASAAACAILSYL”), predicted by SMART as Low Complexity Region (LCR).

**Figure 4:**
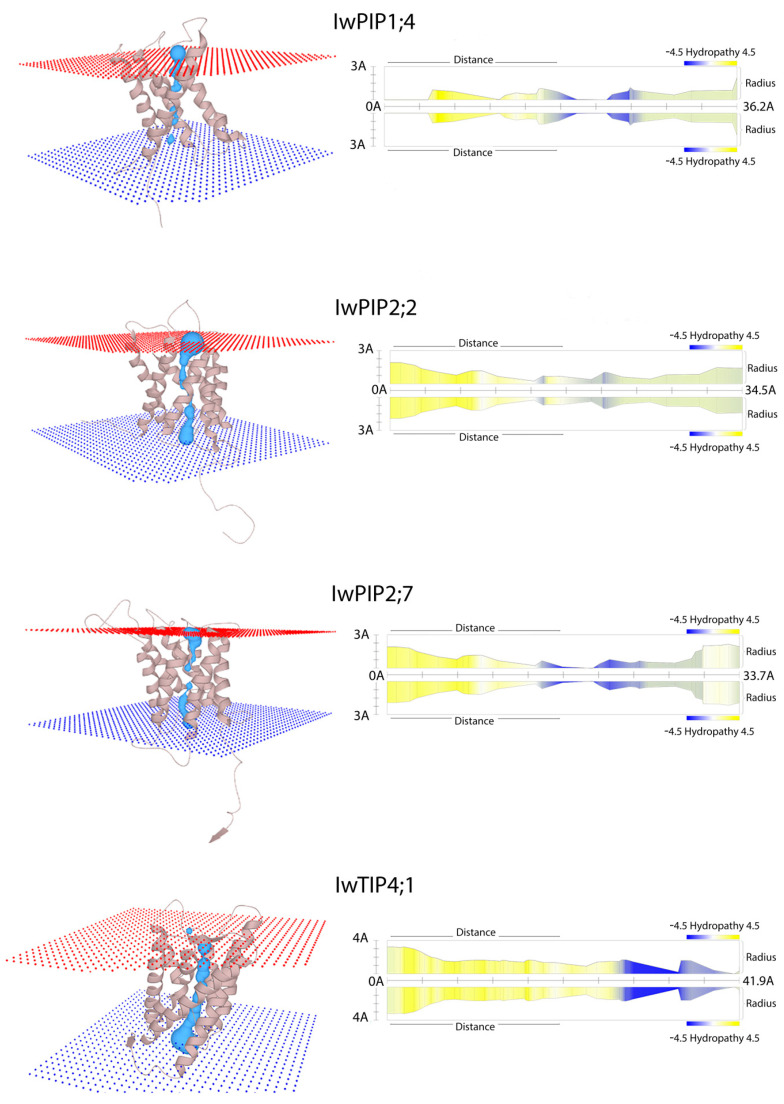
3D structure of pore (blue colored) morphology of individual aquaporins (IwPIP1;4, IwPIP2;2, IwPIP2;7 and IwTIP4;1) monomers in *I. walleriana* (left) with the hydropathy indexes (right), obtained by MOLE2.5 software.

The 3D models generated by PHYRE2 were further analyzed by MOLE2.5 software to study the pore morphology of individual AQPs monomers in the membranes, along with the hydropathy index (Figure 4). The pore morphology of individual aquaporins monomers in the membranes along with the hydropathy index are presented in Figure 4. The results indicate varying composition of the pore amino acid residues in *I. walleriana* aquaporins, which affects pore hydropathy and implies the possibility for diverse solute transport through the aquaporins. The hydropathy index of proteins describes the hydrophobic and hydrophilic properties of amino acids in their sidechain. The hydropathy index of proteins generally has values from −4.5 to 4.5, where a value of 4.5 corresponds to the most hydrophobic amino acid isoleucin, whereas −4.5 corresponds to the most hydrophilic arginine. Additionally, the 3 Å (for IwPIP1;4, IwPIP2;2, IwPIP2;7) and 4 Å diameters (for IwTIP4;1) of the narrowest part of the pores are larger than the ≈2.8 Å diameter of the water molecule. Lengths of aquaporins pores were 36.2 Å, 34.5 Å, 33.7 Å and 41.9 Å, respectively, for IwPIP1;4, IwPIP2;2, IwPIP2;7 and IwTIP4;1.

**Figure 5:**
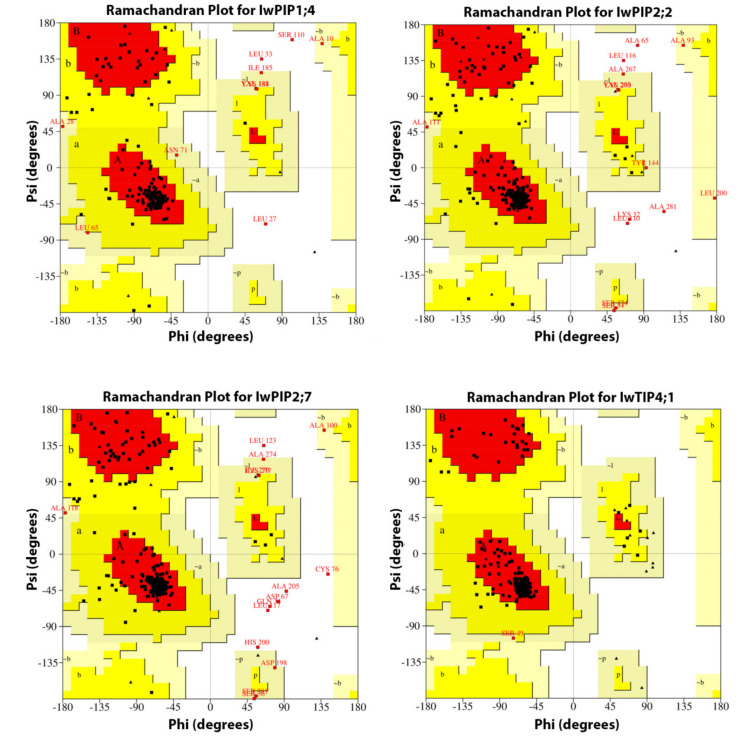
Ramachandran plots for *I. walleriana* aquaporins (IwPIP1;4, IwPIP2;2, IwPIP2;7 and IwTIP4;1 obtained by PROCHECK server. Red = most favored, yellow = additional allowed, cream = generously allowed and white = disallowed. Amino acids in generously allowed and disallowed regions were named.

The quality of *I. walleriana* aquaporins 3D structures generated by PHYRE2 was further assessed by Ramachandran plots (Figure 5). Each amino acid of a protein is characterized by its torsion angles. The torsion angle of the N-Cα bond is called phi and that of the Cα-C bond is psi. The phi-psi angles cluster into distinct regions in the Ramachandran plot, where each region corresponds to a particular secondary structure of proteins. Based on the obtained results, it can be concluded that most amino acids of the analyzed *I. walleriana* aquaporins fall in regions of the Ramachandran plot, which are energetically allowed. These regions included the most favored regions: A region (α helix region), B region (β strands) and L (both types of protein secondary structures). Likewise, energetically allowed regions included residues in additional allowed regions (a, b, l, p) and residues in generously allowed regions (~a,~b,~l,~p). The analysis of calculated Ramachandran plot for IwPIP1;4, IwPIP2;2, IwPIP2;7 and TIP4;1 aquaporins displayed 98.1%, 97.7%, 96.8% and 100% of amino acids in regions that are energetically allowed, respectively (Table 2).

**Figure 6:**
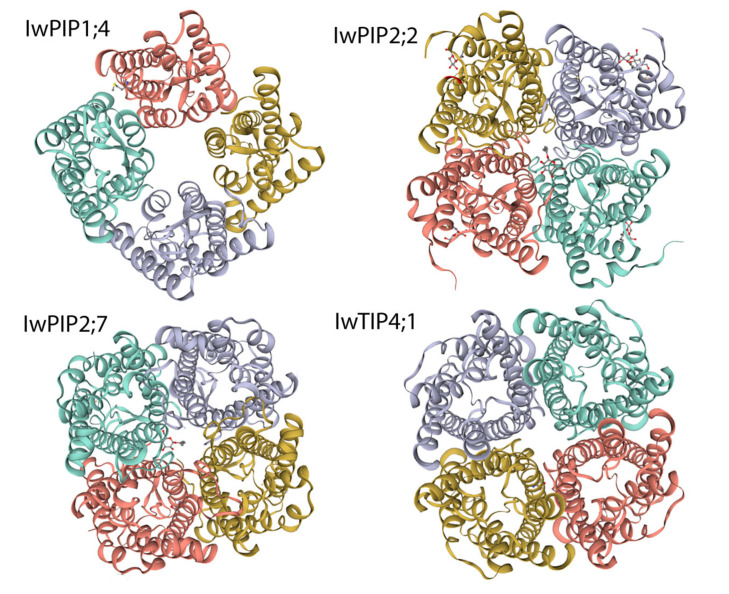
Tetrameric quaternary structures of four *I. walleriana* aquaporins (IwPIP1;4, IwPIP2;2, IwPIP2;7 and IwTIP4;1) generated by SWISS-MODEL.

Structure homology modeling by SWISS-MODEL was used to structurally present the 3D tetrameric form of four aquaporins from *I. walleriana*. All of the *I. walleriana* aquaporins’ modeled structures displayed a high degree of conformity with the crystal structures of aquaporins from other plants (PDB accession number: 4jc6.2.D, 6qim.1.A, 5i32.1.A and many others) with four united monomers forming tetrameric structure of holoproteins (Figure 6). Central pore for water transport formed by two NPA motifs located in two half-TMs is noticeable in all individual monomers of *I. walleriana* aquaporins. In addition to the four individual monomer pores, a fifth pore at the center of the aquaporin tetramer is also presented in the tetramers structures of *I. walleriana* aquaporins.

### 2.2. Aquaporins Expression in Drought-Stressed and Recovered I. walleriana

**Figure 7:**
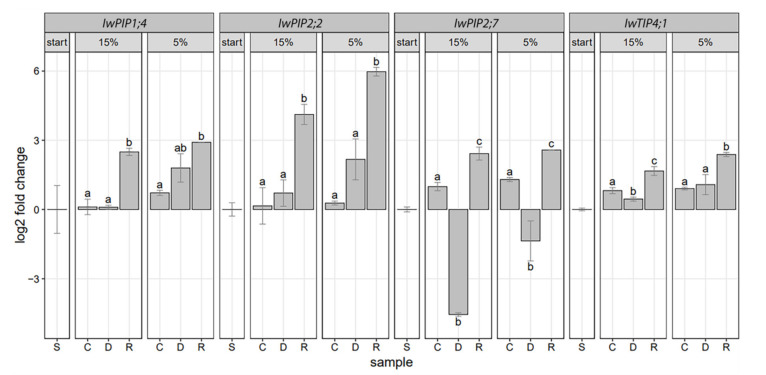
The effect of drought stress and recovery on *IwPIP1;4*, *IwPIP2;2, IwPIP2;7* and *IwTIP4;1* relative gene expression in *I. walleriana* grown ex vitro. Treatments are labeled as S—the “start point” of drought stress imposition, C—control, D—drought, R—recovery; Relative expression of four *I. walleriana* aquaporins is determined by quantitative real-time polymerase chain reaction (RT-qPCR), normalized to the housekeeping gene actine and calculated relative to start. Significant differences between treatments (*p* < 0.05) are indicated by a letter above the bars.

Gene expression analysis of four aquaporins from *I. walleriana* showed different pattern of expression in drought stress and recovery conditions. Results indicated that *IwPIP1;4* was slightly upregulated in severe drought as well as in recovered plants from both drought points. (Figure 7). Recovery from the first drought point induced *IwPIP1;4* more than 2-fold in comparison to control and drought-stressed plants, while recovery from the second drought point had a more moderate effect on *IwPIP1;4* expression. Expression of *IwPIP2;2* was of significance only in plants recovered from drought, where it was upregulated. On the other hand, *IwPIP2;7* was strongly downregulated at the first drought point, while the second drought point moderately downregulated *IwPIP2;7* expression. Recovery treatment from both drought points affected *IwPIP2;7* expression by upregulation. The changes of *IwTIP4;1* in response to drought stress and recovery were very subtle. Tonoplast aquaporin was slightly downregulated at the first drought point and up-regulated in plants recovered from drought stress.

**Figure 8:**
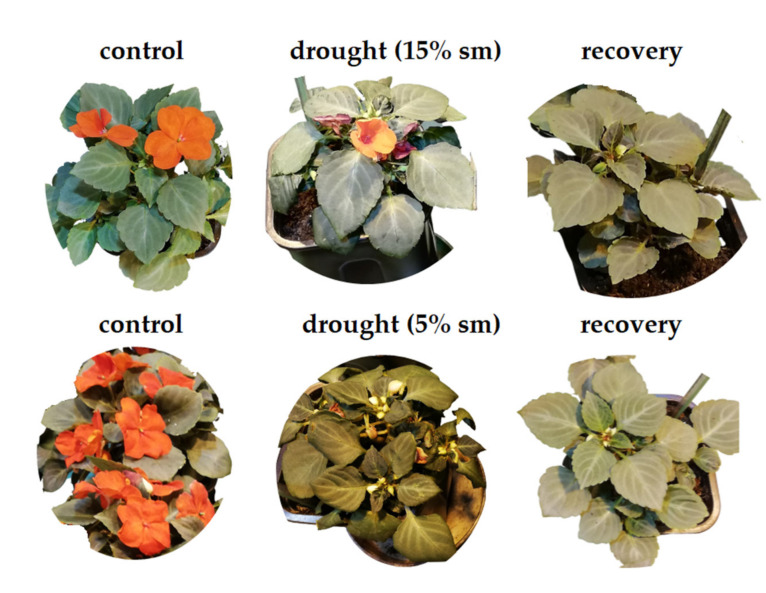
Effect of drought and recovery on *I. walleriana* grown ex vitro. Control, drought-stressed (15% and 5% sm—soil moisture) and recovered plants are labeled.

Morphological differences between *I. walleriana* after stress and recovery are shown in Figure 8. Results indicating that the disturbed water uptake and transport through the cells in drought-stressed plants affected their growth, which was reduced in comparison to control and recovered plants.

## 3. Discussion

Due to their predominant role in the transport of water and other solutes, aquaporins are extensively studied in various plant species. Phylogenetic analysis reveals that *I. walleriana* aquaporins belong to PIP1, PIP2 and TIP4 subgroups, while the analysis of *I. walleriana* aquaporins’ protein sequences provided insight into their molecular structure. The molecular structure of analyzed *I*. *walleriana* aquaporins corresponds to previously reported aquaporins structures in other plant species [24,25,26,27,28]. The theoretical protein pI of *I. walleriana* aquaporins is correlated with their functional location. Proteins of cytoplasmic or vacuolar origin, such as IwTIP4;1, had a lower pI, whereas plasma membrane proteins (IwPIP1;4, IwPIP2;2, IwPIP2;7) had a higher pI. The instability index classified the *I. walleriana* aquaporins proteins as stable. All of the studied *I. walleriana* aquaporins contained the expected dual NPA motifs. Three *I. walleriana* aquaporins with complete coding regions contained the expected six transmembrane helices, while IwPIP1;4, which had partial coding sequence, contained five TM regions. Additionally, one LCR region in IwTIP4;1 was detected. LCRs are regions with little diversity in amino acid sequences and low information content [29]. They are extremely abundant in eukaryotic proteins but research on LCRs’ function in proteins is still deficient [29,30,31]. There is not much information about the presence and role of LCRs in plants, but their role in human diseases has been described [31]; namely, uncontrolled expansion of LCR regions could lead to self-aggregation and formation of amyloid fibrils that cause several human diseases, including Type II diabetes, rheumatoid arthritis and several progressive neurodegenerative disorders such as Alzheimer’s disease, Parkinson’s disease, Spinocerebellar ataxias and Huntington’s disease [31,32,33]. The obtained 3D models of *I. walleriana* aquaporins monomers in membranes with marked pores for water transport are consistent with recent research [28]. The quality of the obtained structures was assessed by Ramachandran plots which indicated that a relatively low percentage of residues have phi/psi angles in the disallowed regions, which suggests the prospective acceptability of Ramachandran plot for *I. walleriana* aquaporins. The quality of protein structure is acceptable if its amino acids fall in regions of the Ramachandran plot that are energetically allowed [34,35,36]. Our findings are consistent with many literature data reported previously. Stereochemical properties of crucin protein in *Jatropha curcas* indicated that 96.30% of the residues were placed into the energetically allowed regions (84.0%, 12.3%, 1.9% and 1.9% of the residues were in the most favored, additional allowed, generously allowed and disallowed regions, respectively) [37]. Protein from *Jatropha curcas* had slightly higher stereochemical quality because only five amino acids had a disallowed geometry. Recently reported analysis of DREB transcription factors (DREB1A, DREB1B and DREB1C) in *Oryza sativa* suggested that overall, 99.00, 98.40 and 98.60% of amino acids are present in the allowed regions, respectively [38]. Ramachandran plots for *Arachis hypogea* lipoxygenase and hydroperoxide lyase enzymes, as well as for *Glycine max* hydroperoxide lyase enzymes predicted by PROCHECK, indicated 77.6%, 65.7% and 82.4% of residues in the most favored regions, respectively. Residues in disallowed regions were 1.1%, 5.7% and 1.2%, respectively, for these three enzymes [39]. PROCHECK Ramachandran plot of the rice Xa21 protein model generated by the HHpred server showed that 99.2% of residues in the model were in the allowed region (78.9% in the most favored, 18.4% in the additionally allowed, 1.9% in generously allowed and 0.8% in disallowed regions). The model generated by PHYRE2 for the same protein had 4% less residues in the most favored regions [40]. Similar results were described for rice cytokinin oxidase/dehydrogenase 2 (CKX2) [41]. Enzyme rice urease, well known for catalyzes the hydrolysis of urea into ammonia and carbon dioxide, has 83.6% of residues in the most favored regions, 15.4% in additionally allowed, 0.8% in generously allowed and 0.1% in disallowed regions, as checked by PROCHECK [42]. In silico characterization of Heat shock factor (Hsf2) from wheat showed 86% of residues in the most favored regions and 14% in additionally allowed regions [43]. Transcription factor DREB1A wheat has 83.7% residues in the most favored regions, 12.2% in additionally allowed and 4.1% in disallowed regions. In this case, Ramachandran plot of TaDREB1A revealed that nearly all amino acids are in allowed regions, and generated structural information of TaDREB1A has been assigned to the biological functions [44]. The hydrophobic and hydrophilic properties of the amino acids in the protein sequences—hydropathy index [45], provided clearer insight into the *I. walleriana* aquaporins structure. Opening and closing of aquaporins water channel pore also depends on the posttranslational modifications such as phosphorylation/dephosphorylation, as well on pH, cation effects, hormone status and reactive oxygen species [7,46]. Abiotic stress could affect all of the mentioned factors, which influence water transport through aquaporins [1,7,46]. In addition, the in silico generated tetrameric structures of *I. walleriana* aquaporins displayed a high degree of conformity with the crystal structures reported previously for tea plant and tobacco [26,27].

Drought stress affects morpho-anatomical characteristics of plants, plant-water relationship, photosynthesis, respiration, mineral nutrition and hormonal balance [1]. In our previous work [3], we described reduced fresh and dry weight and decreased shoot water potential in drought-stressed *I. walleriana*. Reduced leaf area and accumulated ABA contributed to reduction of transpirational water loss in drought-stressed *I. walleriana,* while the activity of antioxidants had an important role in neutralizing oxidative stress [3]. In order to increase drought tolerance in *I. walleriana,* salicylic acid was exogenous applied, and its effects on growth and development have been assessed by our research team [47,48]. Analysis of aquaporins gene expression indicated a very important role of these proteins in water transport in plants during drought [49,50,51]. The present study shows modifications of *I. walleriana* aquaporins expression as part of the plants’ adaptive response to drought. The magnitude of expression changes depended on drought stress intensity, especially in the case of *IwPIP2;7*. Depending on the drought intensity, aquaporin genes in *I. walleriana* increased or decreased their expression. At the first drought point (15% of soil moisture), expression of *IwPIP2;7* in *I. walleriana* was strongly downregulated, while the recovery had the opposite effect. Downregulation of *IwPIP2;7* at 15% of soil moisture content could be explained as a contribution to an inhibition of water loss from the leaves through minimizing water flow through cell membranes. Higher expression level of *IwPIP2;7* in *I. walleriana* recovered from 15% of soil moisture could be very important in reestablishing water homeostasis after the stress treatment. The second drought point (5% of soil moisture) moderately downregulated *IwPIP2;7* in comparison to control plants, which could also contribute to an inhibition of transpirational water loss. Recovery from 5% of soil moisture content upregulated *IwPIP2;7* in comparison to drought-stressed plants, indicating the ability of plants to recover after severe drought stress through changes in expression of water transporter *IwPIP2;7*. Expression of *AtPIP2;7* in *A. thaliana* roots and seedlings was repressed by salt treatment [46,52] and by drought stress in leaves [53]. On the other hand, overexpression of *AtPIP2;7* in *A. thaliana* and tomato plants contributed to higher hydraulic conductivity levels and survival rates under both normal and drought conditions [54]. Expression of *Phaseolus vulgaris PvPIP2;7* during drought was cultivar-specific, with greater downregulation of *PvPIP2;7* under drought conditions in drought-tolerant Tiber [55]. Hu et al. [56] showed that *MaPIP2;7* expression was significantly upregulated after osmotic, cold and salt treatments in banana. Additionally, overexpression of *MaPIP2;7* in banana improved tolerance to multiple stresses such as drought, cold and salt [57]. Aquaporin gene *IwPIP2;2* was upregulated only in plants recovered from drought stress (15% and 5% soil moisture). Thus, expression patterns could be associated with the process of re-acclimation of plants than for conferring drought tolerance. It has been shown that *PIP2;2* was upregulated in the leaves and roots by water deficit in three coffee species, suggesting the possible involvement of this gene in controlling the water status of plants and also in the recovery of drought-stressed plants [58]. In *A. thaliana, AtPIP2;2* is one of the abundantly expressed aquaporin isoforms in roots. *A. thaliana pip2;2* mutants display defects in hydraulic conductivity despite the expression of a very close homolog *AtPIP2;3,* which shares >96% identity, demonstrating that close aquaporin homologs could not function redundantly even within the same plant [59]. Therefore, *I. walleriana* aquaporins *IwPIP2;2* and *IwPIP2;7* may have evolved with nonredundant functions in different tissues, and further expression analysis in roots will provide additional information about that. Aquaporins *IwPIP1;4* and *IwTIP4;1* had relatively low expression in control *I. walleriana* plants, as well as during drought stress and recovery. The highest expression of *IwPIP1;4* was detected in plants recovered from the first drought point, plants exposed to severe drought and those recovered from severe drought. Severe drought slightly increased *IwPIP1;4* expression, which may be important in maintaining water homeostasis in the leaf cells. The *IwPIP1:4* expression in plants recovered from drought stress, together with *IwPIP2;2* and *IwPIP2;7* expression, could contribute to plant re-acclimation after stress treatment. In *A. thaliana,* drought stress upregulated *AtPIP1;4* in both the roots and aerial parts of the plants [60]. Similar results are observed for *PIP1;4* expression in three *Pyrus* species [61]. Specifically, drought stress during the summer up-regulated *PIP1;4* across the three species and explained as help the plant to cope with water stress, potentially by channeling water to target cells. Expression profile of *IwTIP4;1* indicated its minor contribution in water conservation through slight downregulation at 15% soil moisture, while the expression in recovered plants has a similar pattern as in three other analyzed aquaporin genes. In barley, drought stress upregulated *HvTIP4;1* in the leaves, while the re-watering returned its expression to the level in non-stressed plants [62]. On the other hand, in *Coffea arabica CaTIP4;1,* was downregulated in root tissue during drought [63]. Interestingly, the gene expression of *PvTIP4;1* during drought was cultivar-specific with greater downregulation of these genes in the drought-tolerant cultivar of *Phaseolus vulgaris* [55]. Considering that IwTIP4;1 protein had a larger pore diameter than other analyzed aquaporins in *I. walleriana,* it can also be speculated about its transport of larger molecules than water. The up- or downregulation of aquaporin genes in *I. walleriana* leaves subjected to drought implies that lower or higher expression of these aquaporin genes is beneficial to keep a suitable status of water under stress conditions.

Influence of drought stress and rehydration on *I. walleriana* growth could be observed on plant morphological and physiological levels [3,47]. As shown in Figure 8, as well as in previous research [3], drought stress reduced plant growth, while the recovery had an opposite effect. Reduced plant growth during drought is a consequence of reduced water transport throught the plant cells and probably downregulated aquaporin transporters for water. In this work, we showed that *IwPIP2;7* was the most downregulated aquaporin in drought, while it increased expression together with other analyzed aquaporins in the recovery state. This increment in aquaporin expression in recovery could ameliorate water flow through the cells and in that way improve plant growth. Recovered plants were visually more similar to control than drought-stressed plants, while effects of rehydration on increasing plant fresh weight, dry weight, total leaf area and shoot water potential have been previously reported [3].

Different patterns of aquaporins expression have been described for many plant species as a response to drought stress. In *A. thaliana* was described as increment and decrement in aquaporin gene expression during drought [53]. Later, Vandeleur et al. (2009) [64] pointed to increased expression of *VvPIP1;1* gene in roots of one *Vitis vinifera* cultivar, while in another cultivar, the expression of the same gene was unchanged during drought. There were no changes in the expression of the analyzed *VvPIP2;2* gene during drought in both cultivars of *V. vinifera*. Increased expression of *OsPIP1;2* and *OsPIP2;1* genes in leaves of wild-type *Oryza sativa* subjected to water deficit stress has also been described [65]. In addition to reducing *PvPIP2;7* and *PvTIP4;1* expression, drought also reduced the *PvPIP1;2* and *PvTIP1;1* expression in leaves of two *Phaseolus vulgaris* cultivars, while rehydration had an opposite effect on aquaporins expression [55]. Different expression pattern of four aquaporin genes was observed in roots and leaves of drought-stressed *Zea mays* and *Sorghum bicolor* [66] as well as for eight aquaporin genes in three genotypes of mulberry plants subjected to drought [67]. The most recent papers indicate the importance of aquaporins expression during drought in two *Fragaria x ananassa* cultivars [68], *Populus deltoids* [69] and *Pennisetum glaucum* [51]. Based on the results from this work and literature data, it is clear that different aquaporin isoforms in different plants have specific roles during drought and recovery, which is also reflected in their differential transcriptional regulation.

Insights into *I. walleriana* aquaporins structure and expression response to drought stress and recovery contribute to the knowledge about drought resistance mechanisms in this plant species. This study represents a basis for further research on aquaporins function in *I. walleriana* in different experimental conditions and/or tissues. An interesting topic for further research would be the improvement of *I. walleriana* resistance to stress by manipulation of aquaporins expression.

## 4. Materials and Methods

### 4.1. Aquaporins Sequences Analysis

Aquaporins sequences were obtained from sequenced *I. walleriana* transcriptome (RNA-seq). Details about transcriptome sequencing have been previously described [3,48]. To identify aquaporin sequences in *I. walleriana,* the leaf transcriptome annotated by Trinotate [3,48] was searched for PFAM accession and Interpro accession IPR000425. The obtained hits were further narrowed down to includes PIP and TIP sequences. The Clustal Omega program (https://www.ebi.ac.uk/Tools/msa/clustalo/) was used for sequences alignment and phylogenetic tree construction. To generate the percentage identity heatmap, pairwise sequence alignments were performed without end gap penalties (overlap alignments) using R package Biostrings [70]. Gap open penalty was ten, while gap extend penalty was four. Percent identity (pid) was calculated as 100 * (identical positions)**/**(aligned positions + internal gap positions). The TMHMM Server v. 2.0 (http://www.cbs.dtu.dk/services/TMHMM-2.0/) and SMART program (http://smart.embl-heidelberg.de/) were used to predict TMh. The molecular weights, theoretical pIs and instability index were predicted using the ProtParam tool (http://web.expasy.org/protparam/), while WoLF PSORT (https://wolfpsort.hgc.jp/) was used to predict subcellular localization of the *I. walleriana* aquaporins. For the *I. walleriana* aquaporins pore morphology study, PHYRE2 was used to generate 3D models (PDB files) [71] from amino acids sequences, which were used to elucidate pore characteristics, hydropathy index, physical and chemical properties in MOLE 2.5 software [72]. The PHYRE2 generated 3D structure was further verified by PROCHECK [73]. The PROCHECK program provides the information about the stereochemical quality of a protein structure. The PROCHECK was used to generate Ramachandran plots, and the quality of the structures was computed in terms of % of residues in favored regions (A, B, L), residues in additional allowed regions (a, b, l, p) and residues in generously allowed regions (~a,~b,~l,~p). Automated holoprotein 3D structure building was conducted by SWISS-MODEL services (http://swissmodel.expasy.org/) [74,75,76]. Protein sequences of *I. walleriana* aquaporins were searched, and 3D models were built on the basis of 30, 30, 20 and 56 filtered templates, respectively for IwPIP1;4, IwPIP2;2, IwPIP2;7 and IwTIP4;1.

### 4.2. Experiment Design, RNA Isolation and Reverse Transcription PCR (RT-PCR)

For the gene expression analysis leaf samples of drought-stressed and recovered *I. walleriana* grown ex vitro were used. Experiment design has been previously described by [3]. *I. walleriana* seeds were germinated on plates containing Klasman Potgrond H commercial substrate (temperature 22–25 °C (day)/17 °C (night), photoperiod 16/8 h (day/night), relative humidity 100% and light intensity 250 mmol m^−1^s^−1^ ). After seed germination, plants continued to grow under the same temperature, light intensity and photoperiod, but relative humidity was 55–60%. One-month-old seedlings were transplanted into 13 cm deep plastic pots and irrigated daily to reach an optimal soil moisture of 35–37%. The drought stress was imposed on 44 day-old plants, namely after 14 days of *I. walleriana* growth in plastic pots under optimal watering. Control plants grew under optimal irrigation (35–37% of soil moisture content) during the whole experimental period, while two other plant groups were not irrigated to reach 15% and 5% moisture in the substrate. Nine days were necessary for plants to achieve 15%, and twenty days to get 5% of soil moisture content. There were also recovery plant groups for both drought treatments, where the effects of drought on plants had been gradually neutralized. Recovery of stressed plants was achieved by watering for four days to optimal soil moisture content (35–37%). For the molecular analysis, the fully expanded fifth leaf from the top was sampled from three plants at “start point” (on time of beginning the drying period), control, drought-stressed (at 15% and 5% soil moisture) and recovered plants (seven treatment groups of *I. walleriana*). All samples were frozen in liquid nitrogen and then stored at −80 °C for further analyses. Differences between start and control plants were only in the age. Plants at the start point were 44 days old, while the control plants for both drought treatment were 53 and 64 days old, respectively. Common for the plants from the start point and control plants was the optimal soil moisture content (35–37%).

Total RNA was isolated from *I. walleriana* leaves (100 mg) according to the method [77]. RNA was quantified with a NanoDrop spectrophotometer (NanoPhotometer^®^ N60, IMPLEN, Munich, Germany), and its quality and integrity were estimated by electrophoretic separation on 1.5% agarose gel. To eliminate traces of DNA, RNA was treated with DNase I (Thermo Fisher Scientific, Waltham, MA, USA) at 37 °C for 10 min, according to the manufacturer’s protocol. cDNAs were synthesized in reverse transcription reaction (RT) from 1 µg of total RNA. The reaction mixture for RT, in volume of 21 µL, contained 10 µL of total RNA (0.1 µg/µL), 25 mM MgCl_2_, 1 mM dNTP, inhibitor RNA-asa (20 U/µL), random hexamers (50 µM) and 15 U of MultiScribe^®^ transcriptase.

### 4.3. Quantitative Real-Time PCR (qRT-PCR)

Relative expression of the *I. walleriana* aquaporin gene was measured by quantitative RT-PCR using SYBR green in QuantStudio 3 Real-Time PCR System (Applied Biosystems, Foster City, CA, USA). All details about primer design, reaction conditions for qRT-PCR and standards preparation were previously described by [3]. The expression levels of the tested aquaporin genes were normalized to the housekeeping gene actine and calculated relative to start (S) control according to the ΔΔCt method [78]. The results are presented as log2 transformation of fold changes (log2FC). Gene expression data were statistically processed in R 4.02 [79]. For each gene at each drought severity (15% and 5%), pairwise Welch’s *t*-tests [80] were used to estimate the significance of ΔΔCt differences between control (C), dehydration (D) and recovery (R) points. Obtained *p*-values were adjusted jointly for all comparisons using the FDR method [81].

Initially, two IwPIP1 (IwPIP1;1 and IwPIP1;3), three IwPIP2 (IwPIP2;2, IwPIP2;4 and IwPIP2;7) and one IwTIP4 (IwTIP4;1) trinity transcripts coding full-length aquaporins, as well as one trinity transcript with a partial coding sequence for IwPIP1;4 (190 amino acids), were chosen for expression studies. Primer details for sequences that produced expected PCR products are given in Table 3. Amplification of actin as a housekeeping referent gene was carried out in parallel with the primers enclosed in Ref. [3].

## 5. Conclusions

Aquaporins play a very important role in plant physiology, and research in recent years has provided a clearer insight into their molecular structure and function. In this research, we have identified four aquaporins from *I. walleriana* transcriptome. Phylogenetic relations to aquaporin sequences from other plants suggest that these sequences belong to the PIP1, PIP2 and TIP4 subgroups. In silico studies of these sequences, including 3D models of the pores show that the identified *I. walleriana* aquaporins correspond to aquaporin structures from other plant species. Since the role of aquaporins is to transport water across the plant membranes in accordance with the physiological state of the organism, we investigated aquaporin gene expression in response to drought and drought recovery. The expression of *IwPIP2;7* was highly responsive to mild drought stress and recovery from mild drought, as well as moderate to severe drought, indicating that *IwPIP2;7* most of all analyzed aquaporins is implicated to drought resistance mechanisms in *I. walleriana.* In rehydration, all analyzed *I. walleriana* aquaporins could have an important role for the cell-to-cell water flow improvement.

## Figures and Tables

**Table 1:** Characteristics of *I. walleriana* aquaporin proteins.

Protein Name	Nucleotide Length (bp)	Amino Acid Number	Coding Sequence (CDS)	MW (kDa)	pI	II	Subcellular Localization
IwPIP1;4	673	190	partial	20.76	9.51	22.08	Plasma membrane
IwPIP2;2	1280	283	complete	30.05	8.22	26.50	Plasma membrane
IwPIP2;7	1302	286	complete	30.51	8.61	26.68	Plasma membrane
IwTIP4;1	1010	248	complete	26.39	5.91	21.26	vacuole

**Table 2:** Stereochemical properties of amino acids in IwPIP1;4, IwPIP2;2, IwPIP2;7 and IwTIP4;1.

Protein Name	A, B, L	a, b, l, p	~a,~b,~l,~p	Disallowed Regions (%)	Energetically Allowed (%) Ʃ
IwPIP1;4	81.5	12.1	4.5	1.9	98.1
IwPIP2;2	79.9	13.6	4.2	2.3	97.7
IwPIP2;7	80.3	12.8	3.7	3.2	96.8
IwTIP4;1	91.8	7.7	0.5	0	100

**Table 3:** Primer sequences characteristic for *I. walleriana* aquaporins.

Gene Nme	Accesion Number	Primer Sequence	Ta (°C)	Amplicon Length (bp)
*IwPIP1;4*	MW316882	FW 5′-ACACTCTTCTGAAAGGCGG-3′R 5′-AGACCCAGTGATCGTTCCAG-3′	60	300
*IwPIP2;2*	MW316883	FW 5′-AGCCGTTGAAGATCATGGGTTA-3′R 5′-CAATCCCTCCAAATCAATACCCT-3′	60	136
*IwPIP2;7*	MW316884	FW 5′-TGGGTTGCTCTGTTCTGTCA-3′R 5′-GTGGGTCGTGGTAGTCCTTG-3′	60	136
*IwTIP4;1*	MW316885	FW 5′-GCGAGTCCACCTCCGATTAG-3′R 5′-CGATGAATCCCGCAAGGTCT-3′	60	100

## Data Availability

All the data are in the manuscript.

## References

[1] Salehi-Lisar S.Y., Bakhshayeshan-Agdam H. (2016). Drought stress in plants: Causes, consequences, and tolerance. Drought Stress Tolerance in Plants.

[2] Waseem M., Ali A., Tahir M., Nadeem M.A., Ayub M., Tanveer A., Ahmad R., Hussain M. (2011). Mechanism of drought tolerance in plant and its management through different methods. Cont. J. Agric. Sci..

[3] Ðurić M., Subotić A., Prokić L., Trifunović-Momčilov M., Cingel A., Vujičić M., Milošević S. (2020). Morpho-Physiological and Molecular Evaluation of Drought and Recovery in *Impatiens walleriana* Grown Ex Vitro. Plants.

[4] Afzal Z., Howton T., Sun Y., Mukhtar M. (2016). The roles of aquaporins in plant stress responses. J. Dev. Biol..

[5] Abascal F., Irisarri I., Zardoya R. (2014). Diversity and evolution of membrane intrinsic proteins. BBA Gen. Subj..

[6] Maurel C., Boursiac Y., Luu D.T., Santoni V., Shahzad Z., Verdoucq L. (2015). Aquaporins in plants. Physiol. Rev..

[7] Kapilan R., Vaziri M., Zwiazek J.J. (2018). Regulation of aquaporins in plants under stress. Biol. Res..

[8] Pawłowicz I., Masajada K. (2019). Aquaporins as a link between water relations and photosynthetic pathway in abiotic stress tolerance in plants. Gene.

[9] Hove R.M., Bhave M. (2011). Plant aquaporins with non-aqua functions: Deciphering the signature sequences. Plant Mol. Biol..

[10] Yaneff A., Vitali V., Amodeo G. (2015). PIP1 aquaporins: Intrinsic water channels or PIP2 aquaporin modulators?. FEBS Lett..

[11] Fitzpatrick K.L., Reid R.J. (2009). The involvement of aquaglyceroporins in transport of boron in barley roots. Plant Cell Environ..

[12] Vajpai M., Mukherjee M., Sankararamakrishnan R. (2018). Cooperativity in plant plasma membrane intrinsic proteins (PIPs): Mechanism of increased water transport in maize PIP1 channels in hetero-tetramers. Sci. Rep..

[13] Bienert M.D., Diehn T.A., Richet N., Chaumont F., Bienert G.P. (2018). Heterotetramerization of plant PIP1 and PIP2 aquaporins is an evolutionary ancient feature to guide PIP1 plasma membrane localization and function. Front. Plant Sci..

[14] Maurel C. (2007). Plant aquaporins: Novel functions and regulation properties. FEBS Lett..

[15] Kurowska M.M. (2020). TIP Aquaporins in Plants: Role in Abiotic Stress Tolerance. Abiotic Stress in Plants.

[16] Gerbeau P., Güçlü J., Ripoche P., Maurel C. (1999). Aquaporin Nt-TIPa can account for the high permeability of tobacco cell vacuolar membrane to small neutral solutes. Plant J..

[17] Jahn T.P., Møller A.L., Zeuthen T., Holm L.M., Klærke D.A., Mohsin B., Kühlbrandt W., Schjoerring J.K. (2004). Aquaporin homologues in plants and mammals transport ammonia. FEBS Lett..

[18] Srivastava A.K., Penna S., Nguyen D.V., Tran L.S.P. (2016). Multifaceted roles of aquaporins as molecular conduits in plant responses to abiotic stresses. Crit. Rev. Biotechnol..

[19] Zargar S.M., Nagar P., Deshmukh R., Nazir M., Wani A.A., Masoodi K.Z., Agrawal G.K., Rakwal R. (2017). Aquaporins as potential drought tolerance inducing proteins: Towards instigating stress tolerance. J. Proteom..

[20] Jia J., Liang Y., Gou T., Hu Y., Zhu Y., Huo H., Guo J., Gong H. (2020). The expression response of plasma membrane aquaporins to salt stress in tomato plants. Environ. Exp. Bot..

[21] Janssens S.B., Knox E.B., Huysmans S., Smets E.F., Merckx V.S. (2009). Rapid radiation of Impatiens (Balsaminaceae) during Pliocene and Pleistocene: Result of a global climate change. Mol. Phylogenet. Evol..

[22] Fischer E. (2004). Balsaminaceae. Flowering Plants Dicotyledons.

[23] Grey-Wilson C. (1980). Hydrocera triflora, Its Floral Morphology and Relationship with Impatiens: Studies in Balsaminaceae: V. Kew Bull..

[24] Chaumont F., Barrieu F., Jung R., Chrispeels M.J. (2000). Plasma membrane intrinsic proteins from maize cluster in two sequence subgroups with differential aquaporin activity. Plant Physiol..

[25] Dong Y., Yang C., Zhang D., Wang Y. (2007). Cloning and sequence analysis of gene encoding plasma aquaporin of Tamarix albiflonum. Front. For. China.

[26] Yue C., Cao H., Wang L., Zhou Y., Hao X., Zeng J., Wang X., Yang Y. (2014). Molecular cloning and expression analysis of tea plant aquaporin (AQUAPORIN) gene family. Plant Physiol. Biochem..

[27] Sun X., Deng Y., Liang L., Jia X., Xiao Z., Su J. (2017). Overexpression of a PIP1 gene from Salicornia bigelovii in tobacco plants improves their drought tolerance. J. Am. Soc. Hortic. Sci..

[28] Kumar N., Kumawat S., Khatri P., Singla P., Tandon G., Bhatt V., Shinde S., Patil G.B., Sonah H., Deshmukh R. (2020). Understanding aquaporin transport system in highly stress-tolerant and medicinal plant species Jujube (Ziziphus jujuba Mill.). J. Biotechnol..

[29] Kumar M., Kumari B., Kumar R. (2017). Structural Analysis of Low Complexity Regions of Proteins. Can J. Biotech..

[30] Toll-Riera M., Radó-Trilla N., Martys F., Alba M.M. (2012). Role of low-complexity sequences in the formation of novel protein coding sequences. Mol. Biol. Evol..

[31] Kumari B., Kumar R., Chauhan V., Kumar M. (2018). Comparative functional analysis of proteins containing low-complexity predicted amyloid regions. PeerJ.

[32] Michelitsch M.D., Weissman J.S. (2000). A census of glutamine/asparagine-rich regions: Implications for their conserved function and the prediction of novel prions. Proc. Natl. Acad. Sci. USA.

[33] Gunawardena S., Goldstein L.S. (2005). Polyglutamine diseases and transport problems: Deadly traffic jams on neuronal highways. Arch. Neurol..

[34] Hollingsworth S.A., Karplus P.A. (2010). A fresh look at the Ramachandran plot and the occurrence of standard structures in proteins. Biomol. Concepts.

[35] Carugo O., Djinović-Carugo K. (2013). A proteomic Ramachandran plot (PRplot). Amino Acids.

[36] Laskowski R.A., Furnham N., Thornton J.M. (2013). The Ramachandran plot and protein structure validation. Biomolecular Forms and Functions: A Celebration of 50 Years of the Ramachandran Map.

[37] Srivastava M., Gupta S.K., Abhilash P.C., Singh N. (2012). Structure prediction and binding sites analysis of curcin protein of Jatropha curcas using computational approaches. J. Mol. Model..

[38] Donde R., Gupta M.K., Gouda G., Kumar J., Vadde R., Sahoo K.K., Dash S.K., Behera L. (2019). Computational characterization of structural and functional roles of DREB1A, DREB1B and DREB1C in enhancing cold tolerance in rice plant. Amino Acids.

[39] Preeti A., Shaifali S., Kumar P.G., Mani P.D. (2019). Computational characterization of lipoxygenase and hydroperoxide lyase enzymes and Real Time PCR-based expression analysis of their encoding genes in peanut under heat and drought stress. Res. J. Biotechnol. Vol..

[40] Mubassir M.H.M., Naser M.A., Abdul-Wahab M.F., Jawad T., Alvy R.I., Hamdan S. (2020). Comprehensive in silico modeling of the rice plant PRR Xa21 and its interaction with RaxX21-sY and OsSERK2. RSC Adv..

[41] Gouda G., Gupta M.K., Donde R., Kumar J., Vadde R., Mohapatra T., Behera L. (2020). Computational approach towards understanding structural and functional role of cytokinin oxidase/dehydrogenase 2 (CKX2) in enhancing grain yield in rice plant. J. Biomol. Struct. Dyn..

[42] Kumar A., Kumar S., Kumar A., Sharma N., Sharma M., Singh K.P., Rathore M., Gajula M.P. (2018). Homology modeling, molecular docking and molecular dynamics based functional insights into rice urease bound to urea. Proc. Natl. Acad. Sci. India Sect. B Biol. Sci..

[43] Dubey K., Goswami S., Kumar N., Kumar R.R., Niraj R.R.K. (2020). Cloning and in silico characterization of Heat shock factor (Hsf2) from Wheat (*Triticum aestivum* L.). bioRxiv.

[44] Kumar A., Kumar S., Kumar U., Suravajhala P., Gajula M.P. (2016). Functional and structural insights into novel DREB1A transcription factors in common wheat (Triticum aestivum L.): A molecular modeling approach. Comput. Biol. Chem..

[45] Kyte J., Doolittle R.F. (1982). A simple method for displaying the hydropathic character of a protein. J. Mol. Biol..

[46] Pou A., Jeanguenin L., Milhiet T., Batoko H., Chaumont F., Hachez C. (2016). Salinity-mediated transcriptional and post-translational regulation of the Arabidopsis aquaporin PIP2; 7. Plant Mol. Biol..

[47] Antonić D., Milošević S., Cingel A., Lojić M., Trifunović-Momčilov M., Petrić M., Simonović A. (2016). Effects of exogenous salicylic acid on Impatiens walleriana L. grown in vitro under polyethylene glycol-imposed drought. S. Afr. J. Bot..

[48] Antonić D.D., Subotić A.R., Dragićević M.B., Pantelić D., Milošević S.M., Simonović A.D., Momčilović I. (2020). Effects of Exogenous Salicylic Acid on Drought Response and Characterization of Dehydrins in Impatiens walleriana. Plants.

[49] Shekoofa A., Sinclair T.R. (2018). Aquaporin activity to improve crop drought tolerance. Cells.

[50] Avila R.T., Cardoso A.A., de Almeida W.L., Costa L.C., Machado K.L., Barbosa M.L., de Souza R.P., Oliveira L.A., Batista D.S., Martins S.C. (2020). Coffee plants respond to drought and elevated [CO_2_] through changes in stomatal function, plant hydraulic conductance, and aquaporin expression. Environ. Exp. Bot..

[51] Iwuala E., Odjegba V., Sharma V., Alam A. (2020). Drought stress modulates expression of aquaporin gene and photosynthetic efficiency in Pennisetum glaucum (L.) R. Br. genotypes. Curr. Plant Biol..

[52] Boursiac Y., Chen S., Luu D.T., Sorieul M., van den Dries N., Maurel C. (2005). Early effects of salinity on water transport in Arabidopsis roots. Molecular and cellular features of aquaporin expression. Plant Physiol..

[53] Alexandersson E., Fraysse L., Sjövall-Larsen S., Gustavsson S., Fellert M., Karlsson M., Johanson U., Kjellbom P. (2005). Whole gene family expression and drought stress regulation of aquaporins. Plant Mol. Biol..

[54] Li R., Wang J., Li S., Zhang L., Qi C., Weeda S., Zhao B., Ren S., Guo Y.D. (2016). Plasma membrane intrinsic proteins SlPIP2; 1, SlPIP2; 7 and SlPIP2; 5 conferring enhanced drought stress tolerance in tomato. Sci. Rep..

[55] Zupin M., Sedlar A., Kidrič M., Meglič V. (2017). Drought-induced expression of aquaporin genes in leaves of two common bean cultivars differing in tolerance to drought stress. J. Plant Res..

[56] Hu W., Ding Z., Tie W., Yan Y., Liu Y., Wu C., Liu J., Wang J., Peng M., Xu B. (2017). Comparative physiological and transcriptomic analyses provide integrated insight into osmotic, cold, and salt stress tolerance mechanisms in banana. Sci. Rep..

[57] Xu Y., Hu W., Liu J., Song S., Hou X., Jia C., Li J., Miao H., Wang Z., Tie W. (2020). An aquaporin gene MaPIP2-7 is involved in tolerance to drought, cold and salt stresses in transgenic banana (Musa acuminata L.). Plant Physiol. Biochem..

[58] Santos A.B.D., Mazzafera P. (2013). Aquaporins and the control of the water status in coffee plants. Theor. Exp. Plant Physiol..

[59] Javot H., Lauvergeat V., Santoni V., Martin-Laurent F., Güçlü J., Vinh J., Heyes J., Franck K.I., Schäffner A.R., Bouchez D. (2003). Role of a single aquaporin isoform in root water uptake. Plant Cell.

[60] Jang J.Y., Kim D.G., Kim Y.O., Kim J.S., Kang H. (2004). An expression analysis of a gene family encoding plasma membrane aquaporins in response to abiotic stresses in Arabidopsis thaliana. Plant Mol. Biol..

[61] Paudel I., Gerbi H., Zisovich A., Sapir G., Ben-Dor S., Brumfeld V., Klein T. (2019). Drought tolerance mechanisms and aquaporin expression of wild vs. cultivated pear tree species in the field. Environ. Exp. Bot..

[62] Kurowska M.M., Wiecha K., Gajek K., Szarejko I. (2019). Drought stress and re-watering affect the abundance of TIP aquaporin transcripts in barley. PLoS ONE.

[63] Miniussi M., Del Terra L., Savi T., Pallavicini A., Nardini A. (2015). Aquaporins in *Coffea arabica* L.: Identification, expression, and impacts on plant water relations and hydraulics. Plant Physiol. Biochem..

[64] Vandeleur R.K., Mayo G., Shelden M.C., Gilliham M., Kaiser B.N., Tyerman S.D. (2009). The role of plasma membrane intrinsic protein aquaporins in water transport through roots: Diurnal and drought stress responses reveal different strategies between isohydric and anisohydric cultivars of grapevine. Plant Physiol..

[65] Yooyongwech S., Cha-um S., Supaibulwatana K. (2013). Water relation and aquaporin genes (PIP1; 2 and PIP2; 1) expression at the reproductive stage of rice (*Oryza sativa* L. spp. indica) mutant subjected to water deficit stress. Plant Omics.

[66] Hasan S.A., Rabei S.H., Nada R.M., Abogadallah G.M. (2017). Water use efficiency in the drought-stressed sorghum and maize in relation to expression of aquaporin genes. Biol. Plant..

[67] Reddy K.S., Sekhar K.M., Reddy A.R. (2017). Genotypic variation in tolerance to drought stress is highly coordinated with hydraulic conductivity–photosynthesis interplay and aquaporin expression in field-grown mulberry (Morus spp.). Tree Physiol..

[68] Merlaen B., De Keyser E., Ding L., Leroux O., Chaumont F., Van Labeke M.C. (2019). Physiological responses and aquaporin expression upon drought and osmotic stress in a conservative vs prodigal Fragaria x ananassa cultivar. Plant Physiol. Biochem..

[69] Muries B., Mom R., Benoit P., Brunel-Michac N., Cochard H., Drevet P., Petel G., Badel E., Fumanal B., Gousset-dupont A. (2019). Aquaporins and water control in drought-stressed poplar leaves: A glimpse into the extraxylem vascular territories. Environ. Exp. Bot..

[70] Pagès H.A., Gentleman P., DebRoy R.S. (2020). R package version 2.56. 0. Biostrings: Efficient Manipulation of Biological Strings.

[71] Kelley L.A., Mezulis S., Yates C.M., Wass M.N., Sternberg M.J. (2015). The Phyre2 web portal for protein modeling, prediction and analysis. Nat. Protoc..

[72] Sehnal D., Vařeková R.S., Berka K., Pravda L., Navrátilová V., Banáš P., Ionescu C.M., Otyepka M., Koča J. (2013). MOLE 2.0: Advanced approach for analysis of biomacromolecular channels. J. Cheminform..

[73] Laskowski R.A., MacArthur M.W., Thornton J.M. (2006). Procheck: Validation of Protein-Structure Coordinates.

[74] Bertoni M., Kiefer F., Biasini M., Bordoli L., Schwede T. (2017). Modeling protein quaternary structure of homo-and hetero-oligomers beyond binary interactions by homology. Sci. Rep..

[75] Bienert S., Waterhouse A., de Beer T.A., Tauriello G., Studer G., Bordoli L., Schwede T. (2017). The SWISS-MODEL Repository—new features and functionality. Nucl. Acids. Res..

[76] Waterhouse A., Bertoni M., Bienert S., Studer G., Tauriello G., Gumienny R., Heer F.T., de Beer T.A.P., Rempfer C., Bordoli L. (2018). SWISS-MODEL: Homology modelling of protein structures and complexes. Nucl. Acids Res..

[77] Gasic K., Hernandez A., Korban S.S. (2004). RNA extraction from different apple tissues rich in polyphenols and polysaccharides for cDNA library construction. Plant. Mol. Biol. Rep..

[78] Livak K.J., Schmittgen T.D. (2001). Analysis of Relative Gene Expression Data Using Real-Time Quantitative PCR and the 2−ΔΔCT Method. Methods.

[79] R Core Team (2020). R: A Language and Environment for Statistical Computing.

[80] Welch B.L. (1947). The generalization of student’s’ problem when several different population variances are involved. Biometrika.

[81] Benjamini Y., Hochberg Y. (1995). Controlling the false discovery rate: A practical and powerful approach to multiple testing. J. R. Stat. Soc. Series B Stat. Methodol..

